# The Bacteria

**Published:** 1881-02

**Authors:** 


### BIBLIOGRAPHICAL.

THE BACTERIA. By Dr. Antoine Maguire, Licentiate of Natural Sciences, Chief of the Practical Labors in Natural History to the Faculty of Medicine of Lyons, etc , etc. Translated by George M. Steinberg, M D., Surgeon U. S. A. Little, Brown & Co., Publishers. Boston. 1880.

The already extensive and growing importance that is assigned to the microscopical organism as associated with various pathological processes, as well as many other chemical alterations, has occasioned much investigation as to their nature and other characteristics.

In the work before us the author deals extensively in a detailed description of bacteria—giving their organization, classification and development. Next is considered the role they play in various normal and abnormal processes.

The work we consider is indispensible as supplementary to studies in pathology or morbid anatomy.

